# Ceftriaxone-induced cholelithiasis in pediatrics: pooled frequency, symptoms, and associated factors — systematic review and meta-analysis

**DOI:** 10.1186/s13052-025-02057-w

**Published:** 2025-10-10

**Authors:** Tahneem Yaseen, Khurshid Alam, Mohammed Zawiah, Amal K. Suleiman, Amer Hayat Khan

**Affiliations:** 1 Masters of Pharmacy (Clinical Pharmacy), Department of Clinical Pharmacy, School of Pharmaceutical Sciences, Universiti Sains Malaysia, Penang, Malaysia; 2 Department of Pharmacy and Applied Health Sciences, Iqra University, Chak Shahzad Campus, Islamabad, Pakistan; 3 Clinical Pharmacy, Department of Clinical Practice, Faculty of Pharmacy, Northern Border University, Rafha, Kingdom of Saudi Arabia; 4 Department of Pharmacy Practice, College of Clinical Pharmacy, King Faisal University, Hofuf, 31982 Al- Ahsa Kingdom of Saudi Arabia; 5 Department of Clinical Pharmacy, School of Pharmaceutical Sciences, Universiti Sains Malaysia, Penang, Malaysia

**Keywords:** Ceftriaxone, Pediatrics, Cholelithiasis, Symptom burden, Meta-analysis

## Abstract

**Abstract:**

Ceftriaxone is commonly used in pediatric infections, but its association with cholelithiasis poses potential health concerns. To determine the pooled frequency of ceftriaxone-induced cholelithiasis in pediatric patients and identify factors commonly associated with its occurrence. Web of Science, PubMed, Google Scholar, and Scopus were systematically searched until March 2024.Studies reporting ceftriaxone-induced cholelithiasis in pediatric patients (0–18 years) were included. Randomized controlled trials (RCTs) and prospective and retrospective cohort studies published in English were eligible. PRISMA guidelines were followed. The Newcastle‒Ottawa Scale and CASP tools were used to assess risk of bias. A random-effects meta-analysis estimated the pooled frequency. Sensitivity analysis was conducted to explore heterogeneity. The primary outcome was the pooled frequency of ceftriaxone-induced cholelithiasis. Secondary outcomes included identification of factors commonly associated with its occurrence and their impact on symptom burden. Eleven studies (1 RCT, 10 cohort studies) met the inclusion criteria. The pooled frequency of cholelithiasis was 15% (95% CI: 9-23%), with significant heterogeneity (I² = 81.76%). Commonly associated factors included high ceftriaxone doses (> 2 g/day), prolonged use (> 5 days), short bolus injections, and dehydration. Most cases resolved upon discontinuation, but symptomatic patients experienced nausea, vomiting, and abdominal pain. Ceftriaxone-induced cholelithiasis is relatively common in pediatric patients, particularly those with associated risk factors. Clinicians should monitor for biliary complications and consider alternative treatments when feasible.

**PROSPERO registration:**

CRD42024503807.

**Supplementary Information:**

The online version contains supplementary material available at 10.1186/s13052-025-02057-w.

## Main text

A meta-analysis of 11 studies revealed a pooled cholelithiasis frequency of 15% (95% CI: 9-23%) among pediatric patients treated with ceftriaxone. Commonly Associated factors included high-dose ceftriaxone (> 2 g/day), prolonged therapy (> 5 days), rapid bolus injections, reduced oral intake, and prior medical conditions. Most cases resolve spontaneously, but symptomatic patients experience temporary Symptom burden disruptions. Ceftriaxone use in pediatric patients carries a significant risk of cholelithiasis, particularly under specific conditions. Clinicians should monitor high-risk children and adjust therapy as needed to minimize complications.

## Introduction

Ceftriaxone, a third-generation cephalosporin, is commonly used in pediatrics due to its broad-spectrum action and effectiveness against key infections. IDSA recommendations for infants and children recommend it for Streptococcus pneumoniae infections, especially in locations with significant penicillin resistance or severe cases like empyema. It is also effective against Haemophilus influenzae, including β-lactamase-producing strains. Its β-lactamase stability makes it an effective first-line therapy for severe bacterial infections in hospitalized children [1].

Because of its high penetration in the cerebrospinal fluid, even when the meninges are not inflamed, it is now commonly used as a simpler alternative to cefotaxime in the treatment of meningitis caused by organisms other than Listeria monocytogenes and faecal streptococci (enterococci) [2]. It is commonly used in neonates, infants, and children to treat sepsis and meningitis [3]. However, prolonged ceftriaxone use is linked to the development of cholelithiasis and pseudolithiasis, particularly in pediatric patients. Ceftriaxone-calcium complexes can precipitate in the biliary system, forming sludge or stones, especially when therapy extends beyond five days [4, 5]. According to epidemiological research, between 17% and 33% of pediatric children receiving treatment develop ceftriaxone-associated biliary sludge or pseudolithiasis. These results are derived from prospective ultrasonographic evaluations carried out both during and following ceftriaxone therapy [6].

In one trial, 118 children were given ceftriaxone at a dose of 100 mg/kg/day. Of these, 17% experienced biliary pseudolithiasis or sludge, which all went away on their own two weeks after the medication was stopped. By the tenth day of treatment, 31% of the 114 pediatric patients in another research had biliary precipitation, with 18% having sludge and 13% having stones [7].

This phenomenon is associated with altered bile composition, the strong affinity of ceftriaxone for calcium, and hepatic transporter variations, particularly in younger patients, where MRP2 and OATP expression continues to mature [8–10]. While often asymptomatic, these conditions can cause abdominal pain and vomiting, potentially affecting a child’s health and requiring timely detection and management [11, 12]. The aims of this systematic review are as follows:

1. The prevalence of cholelithiasis in pediatric patients receiving ceftriaxone was investigated (meta-analysis).
2. Assess commonly associated factors for ceftriaxone-induced cholelithiasis (Systematic Review).
3. Evaluate the symptom burden associated with ceftriaxone-induced cholelithiasis in affected children (Systematic Review).

Hence, the problem statement is as follows:

Although ceftriaxone-associated cholelithiasis in children has been reported in literature, the true prevalence, risk factors, and symptom burden remain unclear. This systematic review and meta-analysis aims to synthesize existing evidence to estimate prevalence, identify key commonly associated factor, and describe the clinical symptoms experienced by affected pediatric patients.

## Methods

This systematic review followed the PRISMA (Preferred Reporting Items for Systematic Reviews and Meta-Analyses) [13] guidelines to ensure methodological rigor and transparency.

This systematic review was conducted in accordance with the PRISMA (Preferred Reporting Items for Systematic Reviews and Meta-Analyses) guidelines. Four electronic databases, PubMed, Scopus, Web of Science, and Google Scholar were systematically searched from inception to March 2024 to identify relevant studies.

The complete table of MeSH terms, Boolean Operators, along with combination of search strings used in the above-mentioned databases is attached in the Table no.1.

**Table 1:** Search strategy adopted for systematic review

	MeSH Terms	Search String
**Drug**	Ceftriaxone	(Ceftriaxone) AND (Cholelithiasis OR Lithiasis OR Gallstone OR “Biliary Sludge”) AND (Pediatrics OR “Pediatric Patients” OR Child OR Children OR Infant OR Adolescent)
**Condition**	Cholelithiasis, Lithiasis, Gallstone, Biliary Sludge	
**Population**	Pediatrics, Pediatric Patients, Child, Children, Infant, Adolescent	

In addition to database searches, supplementary methods were employed to ensure comprehensiveness. These include searching references of included articles. Titles and abstracts were independently screened by two reviewers to determine eligibility, followed by a full-text review. Any discrepancies were resolved through discussion or consultation with a third reviewer. A standardized data extraction form in Excel was used to collect key information, including study design, sample size, population characteristics, diagnostic methods, ceftriaxone dosage and duration, and reported outcomes. Data were independently extracted by two authors, and consistency was checked by a third reviewer. All records were imported into a single EndNote library and deduplicated using the “Find Duplicates” function based on matching titles, authors, and publication details. Records were then exported to Excel with key bibliographic information for screening. Full texts were retrieved using EndNote’s built-in tools, and a final ‘cited by’ search was conducted to ensure no eligible studies were missed.

Risk of bias was assessed using the Newcastle–Ottawa Scale (NOS) [14] for cohort studies as shown in table. No.2. Each study was independently evaluated by two reviewers, and disagreements were resolved by consensus. The NOS evaluates three key domains—selection, comparability, and outcome. Among the included observational studies, NOS scores ranged from 7 to 9 out of 9, indicating fair to good methodological quality; five studies were rated as ‘Good’ (score ≥ 8) and five as ‘Fair’ (score = 7), with the greatest variability observed in the comparability and outcome assessment domains.

**Table 2:** Shows the Bias risk assessment of cohort studies by NOS scale

Study ID	Selection	Comparability	Outcome	Total stars	Classification
**Ustyol. L. 2016**	✵✵✵✵	✵	✵✵✵	8	Good
**Acun. C. 2004**	✵✵✵	✵✵	✵✵	7	Fair
**Alemeyahu. 2014**	✵✵✵	✵✵	✵✵	7	Fair
**Ceran. C. 2004**	✵✵✵	✵✵	✵✵	7	Fair
**Palanduz. A. 2000**	✵✵✵✵	✵	✵✵	7	Fair
**Schaad. 1998**	✵✵✵✵	✵✵	✵✵✵	9	Good
**Bor. 2004**	✵✵✵✵	✵✵	✵✵✵	9	Good
**Ozturk. 2004**	✵✵	✵✵	✵✵✵	7	Fair
**J.P. Bonnet. 1998**	✵✵✵✵	✵✵	✵✵✵	9	Good
**Murata. S. 2015**	✵✵✵✵	✵	✵✵✵	8	Good

**Fig. 1:**
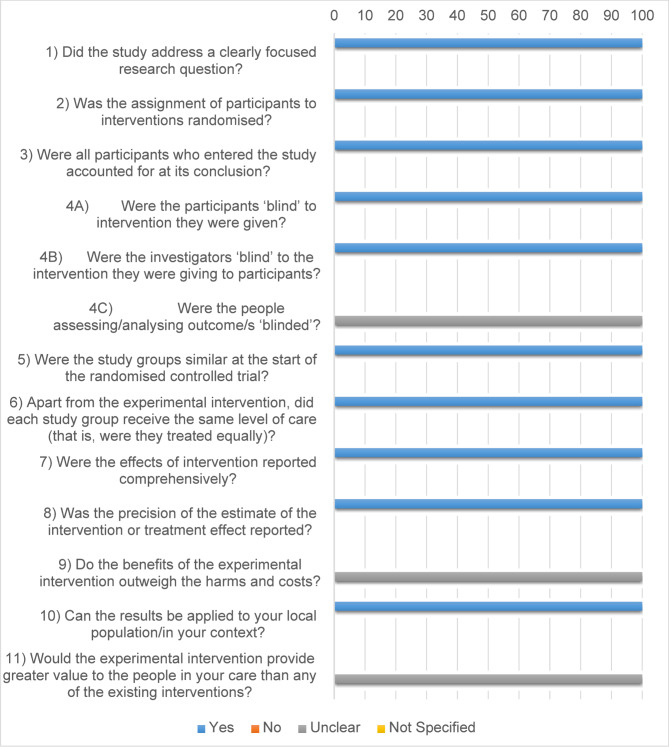
Shows a Stacked bar chart representing the quality of quantitative RCT studies (*n* = 1)

Critical Appraisal Skills Programme (CASP) [15] checklist for randomized controlled trials had (comprises 11 items assessing various aspects of methodological rigor) clearly focused research questions, appropriate randomization, and accounted for all participants; however, blinding of participants, investigators, and outcome assessors was often not adequately reported, introducing some risk of bias. It was performed only for one study as only one RCT was included as shown in Fig. 1.

Although a formal GRADE assessment of certainty was not conducted due to the descriptive nature of the included outcomes, heterogeneity and risk of bias were qualitatively considered when interpreting the pooled results.

The study was registered with PROSPERO (registration number CRD42024503807). There was only one deviation from the original protocol: although the framework was initially based on the PICO (Population, Intervention, Comparator, Outcome) model, the extracted data were more appropriately represented using the PEO (Population, Exposure, Outcome) model, reflecting the descriptive and observational nature of the included studies. The population included pediatric patients (< 18 years), the exposure was ceftriaxone administration, and the outcome of interest was the occurrence of cholelithiasis or biliary pseudolithiasis. Remaining all other objectives and methods remained unchanged and were carried out as pre-specified, in accordance with PRISMA 2020 guidelines.

A preliminary search on PubMed and Google Scholar using the terms **“Ceftriaxone**,** pediatrics**,** cholelithiasis”** identified a relevant systematic review titled **“Ceftriaxone Administration Associated with Lithiasis in Children: Guilty or Not?”** [16] by Louta et al. (2023) examined the potential association between ceftriaxone use and cholelithiasis in pediatric patients, their analysis was descriptive, included case reports and case series, which may introduce bias, and did not perform a meta-analysis or formal evidence grading. In contrast, our study applies the PEO (Population, Exposure, Outcome) framework, which is well-suited for observational data, and conducts a meta-analysis to estimate the pooled prevalence of ceftriaxone-induced cholelithiasis. We included observational studies and RCT to minimize bias, and systematically explore commonly associated factor, including data from case-control studies, as well as assess the impact on symptom burden. Sensitivity analyses were performed to test the robustness of our results. To the best of our knowledge, this is the first meta-analysis to comprehensively quantify the frequency, evaluate specific risk factors, and examine clinical manifestations associated with ceftriaxone-induced cholelithiasis in pediatric patients, highlighting the novelty and clinical relevance of our work.

### Meta-analysis

A random effects model [17] was preselected to compute the pooled proportion of patients with cholelithiasis, along with a 95% confidence interval. The heterogeneity of the frequency of ceftriaxone-induced cholelithiasis between study populations was calculated via Cochran’s Q (heterogeneity T², which is known as the chi square test for heterogeneity) and I² statistics [18]. I² values of less than 25%, 50% to less than 75%, and more than 75% were regarded as evidence of low, moderate, and high levels of inconsistency, respectively [18]. A P value < 0.05 [19] in the forest plot indicates that the results are significant. Sensitivity analysis was performed to detect the effects of each single study on the pooled results when the included studies were excluded one at a time. The leave-one-out method was used to perform sensitivity analysis. Finally, publication bias was analyzed via a funnel plot.

All meta-analyses were conducted using STATA version 18 (StataCorp LLC, College Station, TX, USA). The pooled prevalence of ceftriaxone-induced cholelithiasis was estimated using the metaprop> command under a random-effects model. The metan command was used to generate forest plots, and heterogeneity was assessed using Cochran’s Q and I² statistics. Funnel plots were created using the metafunnel command, and Egger’s test for publication bias was performed using the metabias command [20].

## Results

Searches were completed across all selected databases on the same day for consistency, and the results were exported to EndNote for duplicate removal on the basis of title, author, year, and journal. Additional records from experts, citation tracking, and manual searching were included. Unique references were transferred to Excel with key details such as author, year, journal, DOI, and abstract for screening. EndNote’s “Find Full Text” feature facilitated full-text retrieval. Initial screening of titles and abstracts identified relevant studies, which were further assessed in full text against the inclusion and exclusion criteria. A final “cited by” review ensured comprehensive coverage.

A total of 7,013 records were identified through searches in four databases: Google Scholar (*n* = 6,399), Web of Science (*n* = 502), Elsevier (*n* = 82), and PubMed (*n* = 30). After removing 520 duplicate records, 6,493 unique records remained and were screened based on title and abstract. Of these, 6,256 were excluded for not meeting the inclusion criteria, and an additional 24 were excluded for being published in languages other than English.

**Fig. 2:**
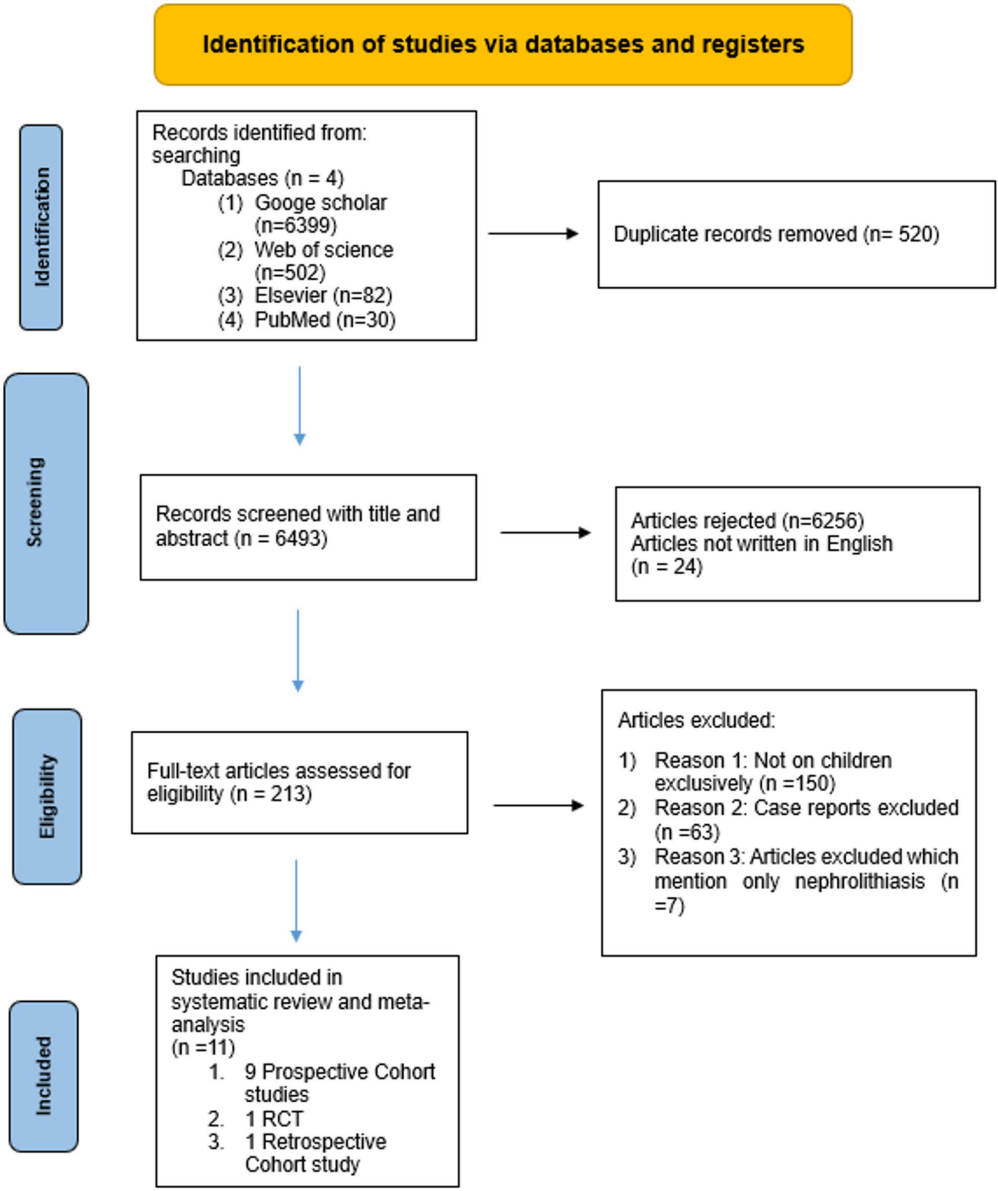
Shows Flow diagram of literature research and article selection process according to the PRISMA guidelines [13]

A total of 213 full-text articles were assessed for eligibility. Among these, 202 articles were excluded for the following reasons: not conducted exclusively in pediatric populations (*n* = 150), case reports (*n* = 63), and studies reporting only nephrolithiasis outcomes without cholelithiasis data (*n* = 7). Ultimately, 11 studies were included in the final systematic review and meta-analysis. These consisted of 9 prospective cohort studies, 1 randomized controlled trial, and 1 retrospective cohort study. The study selection process is illustrated in the PRISMA flow diagram (Fig. 2).

Table 3 in Additional file 1, shows summary of Generalized Main findings from the articles reviewed (*n* = 11).

**Table 3:** Summary of generalized main findings from the articles reviewed (*n*=11)

Author, year	Country	Mean/median of age	Male Number	Female Number	Ceftriaxone dose	Sample size of ceftriaxone users	Sample size of ceftriaxone non-users	Cholelithiasis among ceftriaxone users	Number of lithiasis among ceftriaxone non-users	Study design	Time of occurring the cholelithiasis after using ceftriaxone
BOR et al. 2004	Turkey	62.8±8 months	21	17	100 mg/kg/day, 7 days	38	N/A	13	N/A	Prospective cohort study	10th day of therapy
Murata et al. 2015	Japan	Median: 4 years, infants & children <16 years	23	37	60-100 mg/kg/day	60	N/A	11	N/A	Prospective cohort study	Within 10 days of therapy
SCHAAD et al. 1998	Switzerland	Older (7-8 years), SD 5.4 vs. 24, SD 2-7	21	16	100 mg/kg in one daily intravenous dose	37	N/A	16	N/A	Prospective cohort study	4 to 22 days
Palanduz et al. 2000	Turkey	4.6±3.15 years	66	52	100 mg/kg/day	118	N/A	12	N/A	Prospective cohort study	After 5±11 days of treatment
Ceran et al. 2004	Turkey	7.85±4.75 years	13	37	100 mg/kg/day	50	N/A	1	N/A	Prospective cohort study	8th day of treatment
A. OZTURK et al. 2004	Turkey	76.2±54.8 months	19	14	100 mg/kg/day	33	N/A	7	N/A	Prospective cohort study	4th–8th day of treatment
Acun et al. 2004	Turkey	4.08±3.6 years	18	17	100 mg/kg/day, bid	35	N/A	1	N/A	Prospective cohort study	4–9th day of treatment
Bonnet et al. 2000	France	Median age of 6 years	N/A	N/A	50 mg/kg/day	34	N/A	5	N/A	Prospective cohort study	5th day of treatment
Alemayehu et al. 2014	USA	10.8±3.8 years	N/A	N/A	50 mg/kg/day, 8.7±4 days	71	N/A	10	N/A	Retrospective cohort study	11th day of treatment
Soysal et al. 2007	Turkey	3.8±3.9 years	58	56	>2 g OD	114	N/A	15	N/A	RCT	10th day of treatment
Ustyol et al. 2017	Turkey	4.77±4.91 years	76	78	100 mg/kg/day divided into two equal doses	86 (43 males, 43 females)	68 (33 females, 35 males)	13 (Biliary Sludge=4, Biliary lithiasis=0, Nephrolithiasis=2)	N/A	Prospective cohort study	13th day of treatment

### Forest plot

**Fig. 3:**
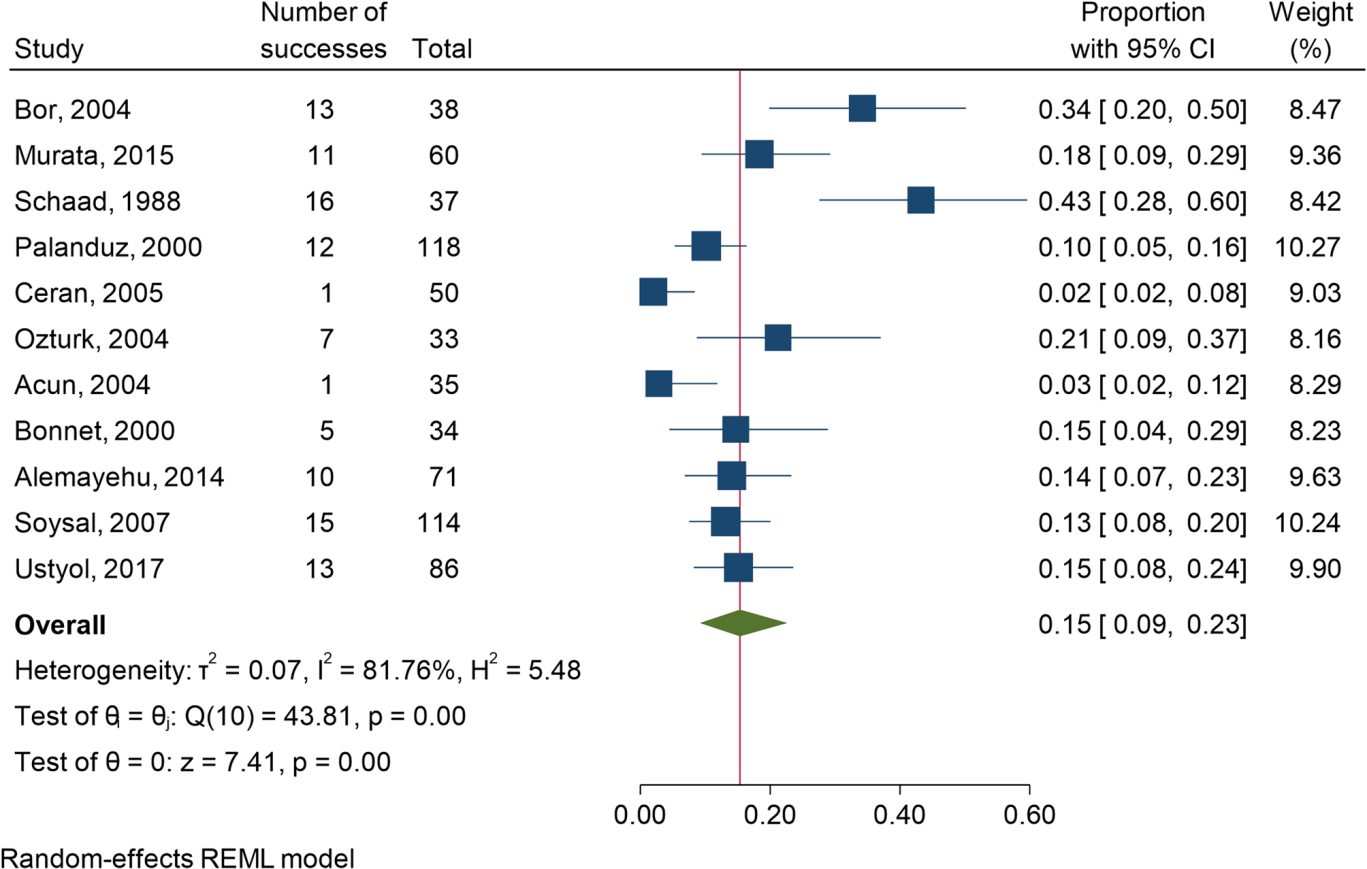
Forest Plot of the Overall Frequency of reported events of cholelithiasis among children using ceftriaxone

Figure 3 shows the number of reported events of cholelithiasis (labeled as “number of successes”) among the total number of participants. The position of the square markers along the x-axis represents the proportion of patients with ceftriaxone-induced cholelithiasis in each study. The diamond at the bottom represents the pooled estimate (overall frequency) of ceftriaxone-induced cholelithiasis across all studies. The pooled proportion is approximately 0.15 (or 15%), with a 95% CI of 0.09–0.23. The plot shows high heterogeneity, indicated by an I² of 81.76%. H² is 5.48, and values above 1 indicate heterogeneity. Therefore, these findings suggest substantial heterogeneity.

The test of θ = θi” (Q test) Q = 43.81 has a p value of 0.00, further confirming significant heterogeneity across studies. The test of θ = 0” shows a z value of 7.41 with a p value of 0.00, suggesting that the overall pooled effect is significant (*p* < 0.05), indicating that the overall proportion of 0.15 is unlikely due to random chance.

Given this high level of variability, sensitivity analyses using the leave-one-out method were conducted to assess the influence of individual studies on the pooled estimate. Although subgroup analysis is a valuable method for exploring heterogeneity, it was not feasible in this review due to limitations in the available data. The included studies exhibited significant variability and often lacked consistent reporting on key stratifying variables such as patient age groups, ceftriaxone dosage (mg/kg/day), treatment duration, route of administration (e.g., bolus vs. infusion), and hydration status. Conducting subgroup analyses without standardized, comparable data would risk introducing bias and misinterpretation. Therefore, to preserve the methodological integrity of the meta-analysis, we relied on sensitivity analysis and qualitative exploration of heterogeneity instead.

### Sensitivity analysis

**Fig. 4:**
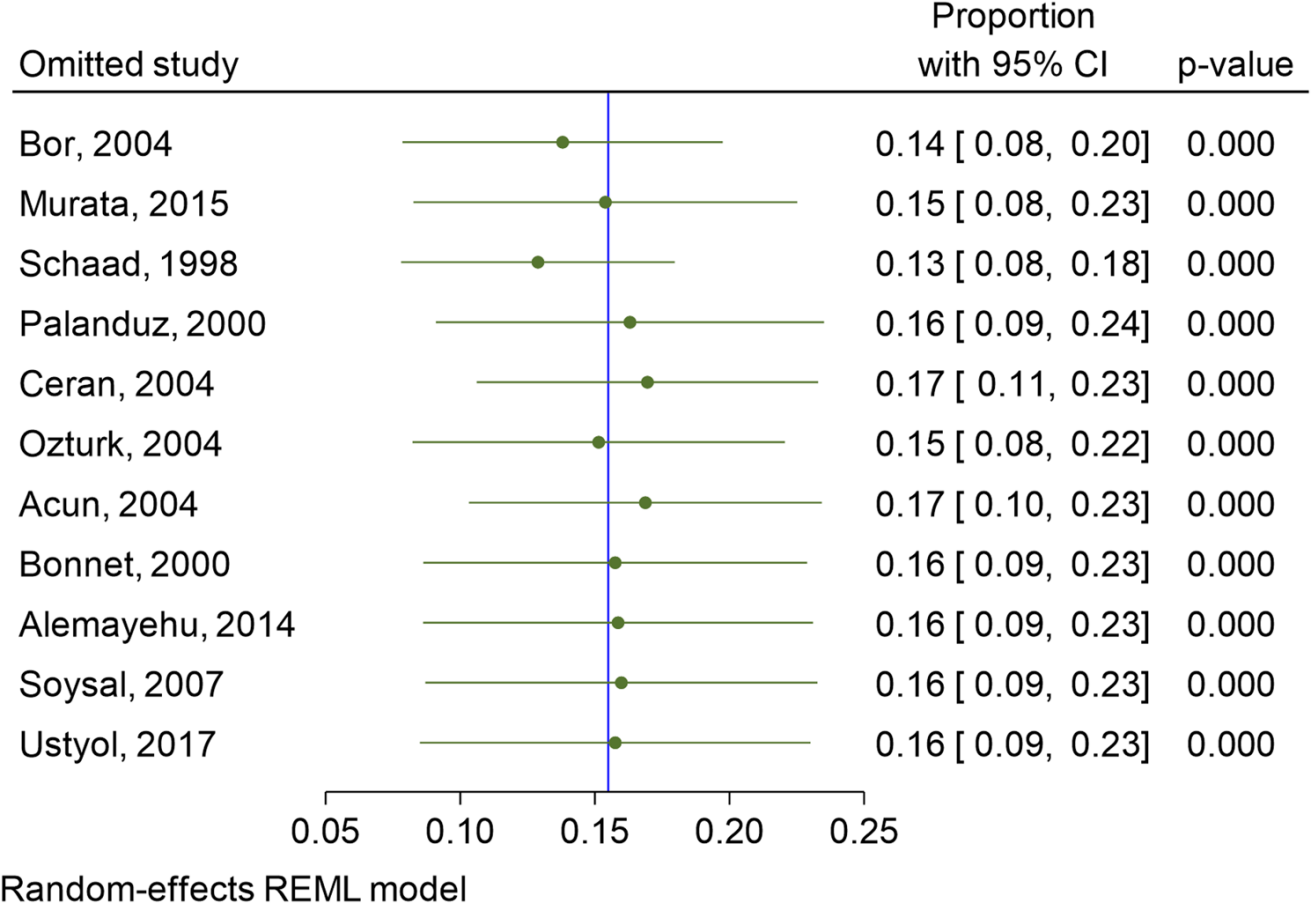
Shows Sensitivity analysis using the Leav-One-Out method

The proportion of patients who developed cholelithiasis across all studies ranged from 0.13 to 0.17, which is a relatively narrow range, indicating that a small percentage of patients consistently experienced this adverse effect, as illustrated in Fig. 4. The pooled estimate of 16% is represented by the blue vertical line in the forest plot. The overall pooled estimate of 16% indicates that, on average, **16 out of every 100 pediatric patients treated with ceftriaxone are expected to develop cholelithiasis**. This estimate is derived by averaging the proportions across multiple studies. All p values are 0.00, which means that the results are statistically significant, and the observed proportions of patients with ceftriaxone-induced cholelithiasis are unlikely to be due to chance.

### Funnel plot

**Fig. 5:**
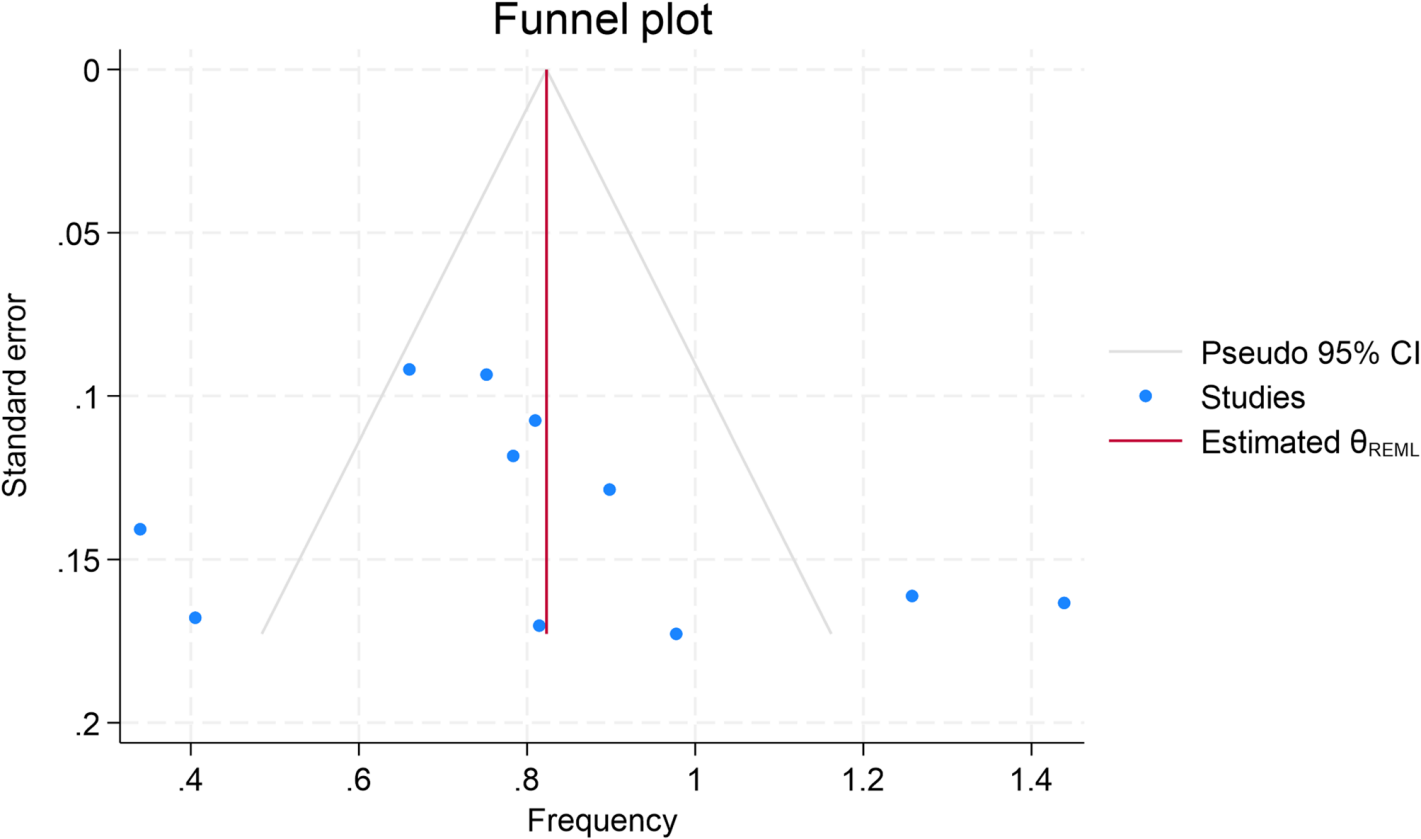
Shows the Publication Bias Plot

As shown in Fig. 5, the funnel plot demonstrates slight asymmetry, with a visual imbalance around the pooled effect size This may be attributed to a combination of random variation, heterogeneity in study designs, and the limited power of statistical tests given the small number of included studies. With only 11 studies in the analysis, caution is warranted in drawing firm conclusions about the absence or presence of publication bias. It is therefore more appropriate to interpret these results as inconclusive rather than definitively symmetric.

While Egger’s test did not indicate statistically significant publication bias (T = 0.920, *p* = 0.383), the reliability of this result must be interpreted with caution due to the small number of included studies. It is well-documented in the meta-analysis literature that the power of Egger’s regression test is substantially limited when fewer than 10–15 studies are analyzed. With only 11 studies in our meta-analysis, the test may fail to detect true asymmetry or small-study effects, even if they exist. Moreover, the visual asymmetry observed in the funnel plot could also reflect inherent heterogeneity in study design, population characteristics, or reporting standards rather than publication bias alone. Therefore, while no significant bias was detected statistically, we acknowledge that the test’s limitations prevent a definitive conclusion, and potential bias cannot be entirely ruled out.

## Discussion

This systematic review examines the relationship between ceftriaxone therapy and cholelithiasis in pediatric patients, synthesizing findings from 11 studies conducted in different countries, including Japan, Switzerland, Turkey, the USA, and France, to provide a comprehensive understanding. To the best of our knowledge, this is the first meta-analysis performed on the incidence of ceftriaxone-induced cholelithiasis.

The included studies reported varying proportions of patients who developed cholelithiasis following ceftriaxone treatment, with estimates ranging from as low as 2% to as high as 43%, as evidenced by the proportions and 95% CIs.

However, the high degree of heterogeneity among the studies (I² = 81.76%) indicates substantial variability in the results, likely due to differences in patient populations, treatment protocols, and study methodologies.

Among the 118 patients whose data were complete in a study by Palanduz et al. [6], 20 (17%) presented sonographic abnormalities: 8 had gallbladder sludge, and 12 had cholelithiasis. These abnormalities appeared 5 to 11 days into treatment and resolved within 3 to 15 days after discontinuation. A prospective cohort study by Murata et al. revealed that cholelithiasis was present in 11 out of 60 patients (18.3%) in this study. The condition was detected via ultrasonography within 3 to 10 days after starting ceftriaxone treatment [21].

The study by Bor et al. found that 36.8% of children on ceftriaxone therapy had abnormal gallbladder sonograms, with 28.9% developing cholelithiasis by day 10 [22]. Ustyol et al.‘s 2017 study showed 20.9% of children in the ceftriaxone group had abnormal biliary findings, including 15.1% with cholelithiasis [23].

Similarly, an RCT conducted by Soysal et al. reported a 13% incidence rate, where 15 out of 114 children were affected by ceftriaxone-associated cholelithiasis [7]. Alemayehu et al. conducted a retrospective cohort study and reported that among 71 patients with a mean age of 10.8 ± 3.8 years who met the inclusion criteria, 10 patients (14%) developed gallbladder stones or sludge during the study. The average duration of ceftriaxone therapy was 8.7 ± 3.8 days, and postantibiotic imaging was conducted an average of 11.5 ± 10.3 days after starting ceftriaxone [24]. Ozturk et al. [25] reported 7 cases out of 33 patients, whereas Bonnet et al. [26] reported only 5 cases out of 34 children.

While some studies have shown a strong association between ceftriaxone and lithiasis, some studies have reported a lower incidence rate of cholelithiasis induced by ceftriaxone but a higher incidence of other symptoms of lithiasis, such as the study conducted by Ceran et al. [27], in which out of 50 children, 12 patients (24%) developed mobile, gravity-dependent echogenic material with clear acoustic shadowing, indicating potential gallstones. Additionally, 1 patient (2%) exhibited changes in the echo pattern and fluid level, suggesting biliary sludge. The remaining 37 patients (74%) had normal abdominal sonography findings at the end of their treatment. Similarly, a study by Acun et al. reported only 1 case of biliary lithiasis among the 35 children studied [28]. The study by Bonnet et al. on early biliary pseudolithiasis provided insights into the reversibility of gallstones upon ceftriaxone discontinuation. The normalization of sonographic patterns was consistently observed on the last sonogram performed 2, 3, and 5 months after the discontinuation of ceftriaxone. These findings indicate that the gallstones induced by Ceftriaxone are reversible and that they dissolve spontaneously after the drug is discontinued [26].

### Associated factors

Table 4 in Additional file 2 shows summary of associated factors and symptom burden in pediatric patients by ceftriaxone induced cholelithiasis. Acun et al. highlighted the role of sex, noting a greater prevalence of gallbladder precipitation in females, which may point to underlying hormonal or anatomical predispositions. They also emphasized the critical period between days 4 and 9 of ceftriaxone treatment, during which the risk of developing biliary complications increases. The study suggested that adequate hydration and divided dosing of ceftriaxone could mitigate these risks, stressing the importance of careful management during this vulnerable period [28]. Bonnet et al. reported that a median age of 7 years was a notable factor in the development of gallstones in children treated with ceftriaxone. The study revealed that ceftriaxone, which is often administered at 50 mg/kg daily, could lead to an increased bile concentration of the drug, fostering an environment conducive to stone formation [26]. Alemayehu et al. focused on ceftriaxone use as the primary predisposing factor for pseudolithiasis but did not find a significant correlation between treatment duration and the development of the condition [24]. A prospective study conducted by Murata et al. explored lifestyle factors influencing biliary pseudolithiasis during ceftriaxone treatment. This study investigated the effects of the dosage and administration methods of ceftriaxone. A high dose of ceftriaxone (> 100 mg/kg/day) and a bolus infusion (< 30 min) are more likely causes of ceftriaxone-induced lithiasis in children. Dehydration is considered a risk factor for biliary pseudolithiasis. One study revealed that the presence of calcium ions precipitated ceftriaxone within the gallbladder, potentially leading to gallstone formation [21]. Ozturk et al.‘s [25] study on asymptomatic biliary sludge and pseudolithiasis, along with Palanduz et al.‘s [6] findings of sonographic abnormalities in asymptomatic patients, underscored the generally benign nature of these complications. Schaad et al.‘s investigation into both biliary and urinary tract precipitation provides a comprehensive view of reversible complications associated with ceftriaxone. The timely resolution of gallbladder precipitation and the influence of injection methods on pseudolithiasis rates strengthened the argument that lithogenic complications are intrinsic to ceftriaxone treatment but are not indicative of prolonged or irreversible conditions [29]. A randomized control trial by Soysal et al. identified factors associated with biliary precipitation. The study revealed that ceftriaxone frequently causes transient biliary precipitation in children, and the probability of its occurrence increases with age over 12 months, a daily dose of over 2 g, or a treatment duration exceeding five days [7].

**Table 4:** Shows summary of associated factors and symptom burden in pediatric patients by ceftriaxone induced cholelithiasis

Authors	Associated Factors	Effect on Symptom Burden
**Acun et al.**	Risk factors for biliary pseudolithiasis and sludge formation include high-dose ceftriaxone (2 g/day), increased calcium secretion into bile (e.g., hypercalcemia), decreased bile flow (e.g., fasting or total parenteral nutrition), increased ceftriaxone excretion in bile (e.g., renal failure, high-dose, or long-term treatment), and gallbladder stasis in patients recovering from major surgery.	Ceftriaxone treatment can lead to gallbladder and urinary tract issues such as abdominal pain, nausea, vomiting. Symptoms usually resolve on their own. Gallbladder issues appear 4–9 days into therapy; urinary tract problems emerge about 5 days after stopping treatment.
**Bonnet et al.**	N/A	Ceftriaxone can cause reversible gallbladder precipitates resembling gallstones (biliary pseudolithiasis). Symptoms and sonographic abnormalities typically resolve after discontinuation. Incidence varies across studies.
**BOR et al.**	Elevated ionized calcium concentration in bile leads to insoluble salts forming biliary sludge visible on ultrasound.	Ceftriaxone-induced lithiasis can cause right upper quadrant pain, nausea, vomiting. Rare complications include cholecystitis, gallstone pancreatitis, and choledocholithiasis.
**Ceran et al.**	Risk factors include fasting, age >24 months, gallbladder stasis post-surgery, high-dose treatment (2 g), and short bolus infusions (3–5 min).	Pseudolithiasis resolved spontaneously after stopping treatment, contributing minimally to symptom burden.
**Murata et al.**	Fasting and bed rest are risk factors for ceftriaxone-induced pseudolithiasis.	Ceftriaxone-induced lithiasis can lead to gallstone attacks and severe adverse events like cholecystitis and pancreatitis. However, no severe symptoms were reported; treatment discontinued without further intervention.
**Ozturk et al.**	Ceftriaxone dose 100 mg/kg/day; restriction of oral intake and surgery increase risk. Summer season with high temperatures may cause dehydration facilitating sludge and pseudolithiasis. No significant effect of gender, age, or therapy duration. Surgery was a significant risk factor (*p*<0.005).	Cases of biliary sludge and pseudolithiasis were mostly asymptomatic and resolved after stopping ceftriaxone, indicating minimal symptom burden.
**Palanduz et al.**	Conditions increasing calcium secretion into bile such as decreased bile flow (fasting, TPN), increased ceftriaxone excretion (renal failure, high dose, long-term), and gallbladder stasis in post-surgical patients contribute to lithiasis risk.	Patients with pseudolithiasis were asymptomatic and required no treatment.
**SCHAAD et al.**	All 16 patients with biliary and ureteric calculi had a maternal family history of cholelithiasis/nephrolithiasis.	Biliary concretions caused colicky upper abdominal pain, nausea, vomiting. Complete sonographic resolution occurred 2–63 days post-treatment.
**Soysal et al.**	Risk factors: age >12 months, daily ceftriaxone dose >2 g, treatment >5 days.	Ceftriaxone-induced lithiasis caused upper abdominal pain, biliary obstruction, or pancreatitis. Stones resolved spontaneously after drug cessation.
**Alemayehu et al.**	10% of patients developed pseudolithiasis, with one requiring cholecystectomy 18 days after ceftriaxone cessation. Age and therapy duration were not predictive factors. Biliary pseudolithiasis unrelated to therapy duration.	Most cases were asymptomatic; no significant QoL impact reported.
**Ustyol et al.**	High doses and prolonged treatment with ceftriaxone, fasting, dehydration, and increased biliary secretion are key contributors to pseudolithiasis development.	Although often asymptomatic, biliary pseudolithiasis can cause symptoms such as right upper quadrant pain and nausea in some children.

### Impact on symptom burden

Ceftriaxone-associated biliary pseudolithiasis and sludge in pediatric patients generally have a minimal and reversible impact on symptom burden. Most affected children remain asymptomatic, and in symptomatic cases, symptoms such as abdominal pain, nausea, and vomiting resolve shortly after discontinuing ceftriaxone, with no long-term effects or need for intervention. This was consistently observed across multiple studies, including those by Acun et al. [28], Soysal et al. [7], Bonnet et al. [26], Ozturk et al. [25], Ceran et al. [27], and Schaad et al. [7]. These findings highlight the importance of avoiding unnecessary interventions, such as cholecystectomy, and underscore the value of personalized care in managing such cases. These findings suggest that while ceftriaxone-associated biliary complications may lead to transient clinical symptoms, they are typically reversible and self-limiting.

### Heterogeneity and confounding factors

The analysis revealed substantial heterogeneity among the studies (I² = 81.76%) and a significant Q statistic (Q = 43.81, *p* = 0.00), indicating that the variability in the study results stems from factors beyond mere chance. This heterogeneity is likely driven by several confounding factors, which must be carefully considered to accurately interpret the findings.

The wide variation in reported prevalence rates of ceftriaxone-induced cholelithiasis (ranging from 2 to 43%) likely reflects several interrelated factors across studies. First, patient age distribution varied considerably; younger children may have different biliary transporter expression and hydration status compared to adolescents, affecting stone formation risk. Second, ceftriaxone dosage and duration of therapy were inconsistently reported and may differ significantly across settings, with some studies administering > 100 mg/kg/day or prolonged courses beyond five days, both identified risk factors. Third, diagnostic timing and imaging methods also varied, with some studies performing routine post-treatment ultrasonography while others relied on symptomatic triggers for evaluation. These methodological differences, in addition to setting-specific factors like nutritional status and healthcare protocols, likely contributed to the observed heterogeneity in prevalence estimates.

The studies included in the analysis may differ in design, one RCT, while the others are observational. RCTs typically control for confounding variables through randomization, but observational studies might not. In observational studies, we included differences in clinical settings, which could introduce confounding factors, leading to variability in outcomes that are unrelated to the treatment itself. Genetic predispositions and environmental factors, such as diet and lifestyle, vary widely across study populations, influencing outcomes independently of treatment. For example, children from different geographic regions, as we included studies worldwide, may have varying risks for conditions such as cholelithiasis, potentially confounding the relationship between the treatment and the observed outcomes. Additionally, while reviewing the literature, some studies reported outcomes related to nephrolithiasis and urolithiasis, which were excluded from the analysis to maintain a focus on cholelithiasis. Focusing solely on studies that report cholelithiasis may overlook other important outcomes, such as liver function abnormalities or pancreatitis, further confounding the overall assessment of treatment safety and efficacy.

### Strengths

To the best of our knowledge, this is the first meta-analysis to comprehensively evaluate the pooled frequency of ceftriaxone-induced cholelithiasis.

This study adopts a systematic approach was applied to enhance the reliability of findings., including rigorous quality assessments of selected studies using the CASP and NOS, ensuring the reliability and validity of the findings.

Efforts were made to adhere to the PRISMA guidelines to enhance transparency and reproducibility. A random-effects model was employed to address the substantial heterogeneity across studies, thereby providing a more accurate and generalizable overall estimate. A notable strength of this meta-analysis is its systematic evaluation of commonly associated factorscontributing to ceftriaxone-induced cholelithiasis and the subsequent impact on patients’ Symptom burden.

## Conclusion

This study provides a comprehensive evaluation of the relationship between ceftriaxone use and cholelithiasis in pediatric patients, integrating findings from a meta-analysis and systematic reviews. The meta-analysis pooled data from studies conducted between 1998 and 2017 and revealed that approximately 15% of pediatric patients treated with ceftriaxone developed cholelithiasis, with a 95% CI ranging from 9 to 23%. This association was statistically significant, with a p value < 0.01, indicating a strong likelihood that the observed effect was not due to chance. Sensitivity analyses further confirmed the robustness of these results, showing consistent findings even when individual studies were excluded. Symptomatic cases can lead to temporary complications such as abdominal pain, nausea, and vomiting, which can affect patients’ quality of life. Clinicians should weigh these risks when prescribing ceftriaxone and monitor for potential complications to minimize any negative impact on the patient’s quality of life.

### Limitations

This review has several limitations. One notable limitation is that it includes only articles published in English. As a result, potentially valuable studies published in other languages may have been excluded, which could affect the comprehensiveness of our analysis. Additionally, the review is limited by the fact that only one randomized controlled trial is available on this topic. The inclusion of more RCTs would provide more conclusive and reliable insights into the effects of ceftriaxone.

### Future recommendations

Currently, our analysis provides only pooled frequency data, which limits our ability to establish a clear association between ceftriaxone use and cholelithiasis. Future research should include more comparative studies between ceftriaxone users and nonusers. Such studies could provide odds ratios and help develop a more definitive understanding of the association between ceftriaxone and cholelithiasis. Further exploration into related conditions, such as urolithiasis and nephrolithiasis, in the context of ceftriaxone therapy is essential.

## Electronic supplementary material

Below is the link to the electronic supplementary material.

Supplementary Material 1

## Data Availability

Data and materials is available as separate supplementary file.

## Abbreviations

RCT: Randomized controlled trial
PRISMA: Preferred reporting items for systematic reviews and meta-analyses
NOS: Newcastle-ottawa scale
CASP: Critical appraisal skills programme
PEO: Population, exposure, outcome (used for formulating research questions)
PROSPERO: International prospective register of systematic reviews
CI: Confidence interval
