# Supplementary figures and images for: Ceftriaxone-induced cholelithiasis in pediatrics: pooled frequency, symptoms, and associated factors — systematic review and meta-analysis
Source: Ital J Pediatr. 2025 Oct 10;51:284. doi: 10.1186/s13052-025-02057-w (PMC12512820; doi:10.1186/s13052-025-02057-w)

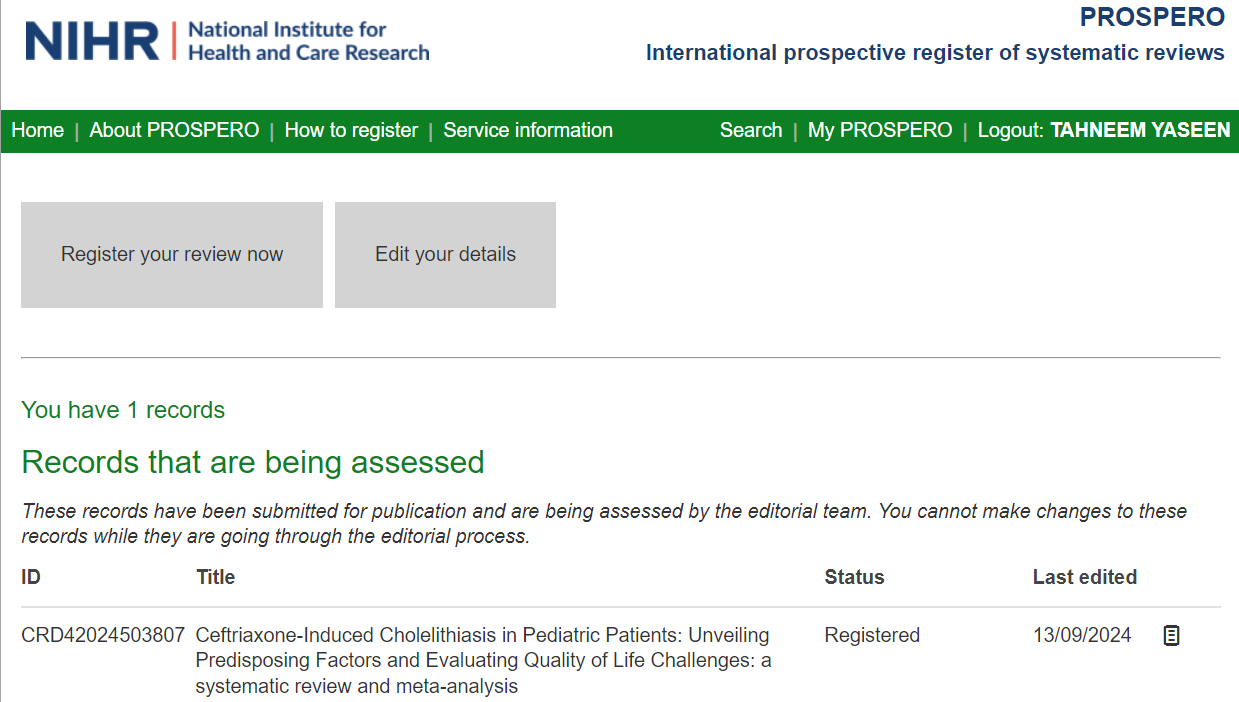

Supplement: Supplementary file 1 — Supplementary Material 1 [file 13052_2025_2057_MOESM1_ESM.docx]
